# “Let’s Talk about OA Pain”: A Qualitative Analysis of the Perceptions of People Suffering from OA. Towards the Development of a Specific Pain OA-Related Questionnaire, the Osteoarthritis Symptom Inventory Scale (OASIS)

**DOI:** 10.1371/journal.pone.0079988

**Published:** 2013-11-11

**Authors:** Christine Cedraschi, Sylvie Delézay, Marc Marty, Francis Berenbaum, Didier Bouhassira, Yves Henrotin, Françoise Laroche, Serge Perrot

**Affiliations:** 1 Multidisciplinary Pain Centre, Division of Clinical Pharmacology and Toxicology & Division of General Medical Rehabilitation, Geneva University Hospitals and University of Geneva, Geneva, Switzerland; 2 Thélaine, Fontenay sous Bois, France; 3 Rheumatology Department, Henri Mondor Hospital, Créteil, France; 4 Rheumatology Department, Saint Antoine Hospital, Paris, France; 5 INSERM U 987, Pain Center, Boulogne, France; 6 Bone and Cartilage Research Unit, University of Liège, Institute of Pathology, CHU, Sart-Tilman, Liège, Belgium; 7 Physical Therapy and Rehabilitation Department, Vivalia, Princess Paola Hospital, Marche-en-Famenne, Belgium; 8 Pain Center, Saint Antoine Hospital, Paris, France; 9 Pain Center, Service de Médecine Interne et Thérapeutique, Hôtel Dieu, Université Paris Descartes, Paris, France; University of South Australia, Australia

## Abstract

**Introduction:**

Pain is the primary outcome measurement in osteoarthritis, and its assessment is mostly based on its intensity. The management of this difficult chronic condition could be improved by using pain descriptors to improve analyses of painful sensations. This should help to define subgroups of patients based on pain phenotype, for more adapted treatment. This study draws upon patients’ descriptions of their pain, to identify and understand their perception of osteoarthritis pain and to categorize pain dimensions.

**Methods:**

This qualitative study was conducted with representative types of patients suffering from osteoarthritis. Two focus groups were conducted with a sample of 14 participants, with either recent or chronic OA, at one or multiple sites. Focus groups were semi-structured and used open-ended questions addressing personal experiences to explore the experiences of patients with OA pain and the meanings they attributed to these pains.

**Results:**

Two main points emerged from content analyses: -A major difficulty in getting patients to describe their osteoarthritis pain: perception that nobody wants to hear about it; necessity to preserve one’s self and social image; notion of self-imposed stoicism; and perception of osteoarthritis as a complex, changing, illogical disease associated with aging. -Osteoarthritis pains were numerous and differed in intensity, duration, depth, type of occurrence, impact and rhythm, but also in painful sensations and associated symptoms. Based on analyses of the verbatim interviews, seven dimensions of OA pain emerged: pain sensory description, OA-related symptoms, pain variability profile, pain-triggering factors, pain and physical activity, mood and image, general physical symptoms.

**Summary:**

In osteoarthritis, pain analysis should not be restricted to intensity. Our qualitative study identified pain descriptors and defined seven dimensions of osteoarthritis pain. Based on these dimensions, we aim to develop a specific questionnaire on osteoarthritis pain quality for osteoarthritis pain phenotyping: the OsteoArthritis Symptom Inventory Scale (OASIS).

## Introduction

In Europe, 20 % of chronic pain is related to osteoarthritis (OA) [1] and pain is the main symptom of OA. Furthermore, OA-related pain is considered to be the prototypical chronic nociceptive pain condition, and is used as a major clinical model for the development of new analgesics for treating chronic pain. Although chronic pain is generally acknowledged to be complex and multidimensional, the assessment of OA pain in clinical trials is mostly one-dimensional, restricted to pain intensity, and in some cases to functional impact or repercussions [2]. 

Melzack [3] introduced three types of pain descriptor, sensory, affective and evaluative, but there are limitations to their use in arthritis [4]. A review of the language used to describe pain indicated that chronic pain experience is multidimensional and individual, but that there are inconsistences in domains between conditions and studies [5]. Some studies have investigated pain descriptors in healthy subjects and chronic pain patients and suggest that 36 words, classified into 12 categories, can be efficiently used to describe pain [6]. These categories and descriptors are widely used in neuropathic pain, with an increasing number of questionnaires developed to assess neuropathic pain quality, including the NPSI [7], the LANNS [8], and PainDetect [9]. These questionnaires have defined several dimensions of neuropathic pain descriptors, and may help to define patient phenotypes likely to benefit from specific analgesic treatments. 

Very few papers have described pain dimensions in OA, and there is little or no consensus concerning these dimensions. Some authors have analyzed night pain in knee osteoarthritis [10] and the prevention of knee pain [11]. A recent initiative from OARSI and OMERACT investigated several dimensions in OA pain, providing ICOAP (Intermittent and Constant OsteoArthritis Pain), a new questionnaire including pain intensity, frequency and impact on mood, sleep, and quality of life [12]. This qualitative approach explored changes in pain characteristics over time, in relation to the priorities and concerns of individuals living with hip or knee pain, using the Patient Generated Index [13], and a Likert index for distress. The authors of this study defined two distinct pain conditions in OA, related to the context of OA progression, with intermittent and intense pain having the greatest impact on quality of life. Several pain descriptors, such as “electrical shock, missing limb, cramps, pulsating knee”, were proposed in the paper, but these descriptors were not included in the ICOAP questionnaire and were not intended for specific use to define pain phenotypes in OA, as already done for neuropathic pain. 

In this context, it was clearly important to gain insight into the patients’ own descriptions of their OA pain, and to consider all the pain sensations described by the patients, to obtain an extensive description of OA pain quality. Indeed, such descriptions should improve evaluation of the various dimensions of OA pain. Our aim was to develop a framework for a patient-centered evaluation of OA pain, allowing an in-depth description of pain, and in further steps, the development of a specific questionnaire, the OsteoArthritis Symptom Inventory Scale (OASIS) for assessing OA pain quality, as a complement to the ICOAP questionnaire, which focuses on OA pain impact. We thus conducted an exploratory qualitative study (i.e. an open-enquiry approach) to investigate how people suffering from OA perceive and describe pain and associated changes.

## Methods

### Study design

Qualitative research methods were used to investigate participants’ perceptions and descriptions of their OA pain [14,15]. These methods made it possible to stress, throughout the interviews, that the goal was to share as much information as possible, as freely as possible, and in the participants’ own words. Focus groups were deemed most appropriate because groups can provide a safe environment in which it is easier for participants to discuss difficulties with their peers [14]. Focus groups tend to make speech easier, since respondents can elaborate on other patients’ input, to enforce a point of view, add nuances to it, or develop an altogether different approach relevant to their own feelings, prompted by seemingly opposite opinions [15,16]. Moreover, group interaction encourages respondents to explore and clarify individual and shared perspectives [15,16].

All subjects were informed of the goals and design of the study and assured of confidentiality before they gave their written informed consent to participate in the study. Data were rendered anonymous to ensure confidentiality. This study was carried out in accordance with the Helsinki Declaration. Approval was obtained from the institutional review board 5Comité Consultatif sur le Traitement de l’Information en Matière de Recherche), and the French data protection agency (Commission Nationale pour l’Informatique et les Libertés) before enrollment. 

Focus groups were conducted by a female PhD psychologist (SD), trained in qualitative procedures, with considerable experience in qualitative studies. No prior relationship with respondents was established before data collection. 

### Participants

Two focus groups were conducted in Paris, France, with 14 respondents. These respondents had suffered severe OA for a number of years, and 10 of them were over 60 years old. Participants were drawn from investigators’ clinical practices, from university hospital and private-practice rheumatologists, and informed about the purpose of the study (i.e to improve our understanding of OA pain). Those eligible to participate were: French-speaking men and women with painful hip, knee or hand OA, who had not experienced joint injury within the last year, or a joint replacement of the symptomatic joint. Anyone with any other type of inflammatory arthritis, fibromyalgia, chronic low back pain, or another chronic pain disorder, such as diabetic neuropathy, was excluded. 

About 80% of the recruited individuals agreed to participate. Those who refused did so mainly because of time contingencies. They did not differ from those who accepted in terms of sociodemographic and clinical characteristics. Respondents were remunerated (€50) for participation. 

Participant selection was intended to ensure that a range of experiences could be investigated; a purposive sample was thus selected, with participants chosen so as to ensure a diverse sample in terms of age, sex and experience and to provide additional insight, based on data from the literature [17,18,19,20]. One of the key advantages of purposive sampling is that it ‘offers a degree of control rather than being at the mercy of any selection bias inherent in pre-existing groups’ [21].

In qualitative studies, the number of participants is usually determined by purposive sampling, i.e. by the need to encompass the range of possible responses [22] and to achieve data saturation [18-20]. Participants are thus selected according to predetermined criteria relevant to the research objective [23]. As the determination of the number of participants required is based on data depth rather than frequencies, the sample should consist of participants as representative as possible of the population studied [24,25]. In this study, data collection essentially addressed the description of the types of pains resulting from OA, rather than focusing on participant’s history and their way of living with pain. Within a pragmatic and flexible approach to sampling [26], a participant sample was included to ensure that the sample was diverse and included experiences of all the types of pains that individuals may experience due to their OA. The use of this participant made it possible to reach a point in data collection and analysis at which new information produced no change in the codebook. The data were therefore considered saturated and this suggests that our sample was sufficiently large to address the research questions considered in this study [25].

### Data collection

The format of the focus groups was semi-structured and used open-ended questions (Table 1) so that the conversation was flexible and responsive, as the moderator carefully explained that osteoarthritis pains can be known and described only by those living with them; all inputs were considered equally relevant and informative, because different patients may have different pains and/or somewhat different perceptions of the same pain. Questions addressed personal experiences and perspectives, to explore the experiences of individuals with OA pain and the meanings they attributed to these pains [16,18]. 

**Table 1:** Interview topic guide.

**Type of question**	** Examples**
**Introduction**	Can you briefly introduce yourself to the group?
**Interview**	Could you please describe all the different types of pains you experience from your osteoarthritis?
	Are there other participants who feel the same?
	Would someone from the group like to add to the description that has just been made?
**Closing**	Is there anything else you would like to add to the list and to the discussion?

Basically, throughout the interviews, participants were asked to “describe as graphically as possible all the different types of pains you experience from your osteoarthritis”. A list was made, each respondent participating by adding one or several types of pain. Each “type of pain” was then described as precisely as possible by the person who first mentioned it, and those who recognized that they “felt the same” then added to the initial description, with specific undertones or sometimes, very small or major differences (starting morning pains were fairly consensual for example, but lasted longer, and were sharper, for some). 

All interviews were taped and transcribed verbatim, with field notes taken during and after participants’ meetings.

### Data analysis

The qualitative analysis began with close readings of the transcripts. On the basis of these transcripts, thematic content analysis led to the identification of categories and themes [27]. Data were analyzed iteratively, to refine evolving themes [19,20,28,29]. Iteration between analyses and the development of categories continued until additional observations provided no new information changing the categories any further [18,19,20]. These categories served as the basis for a final grid, which was then used to analyze the transcripts. The sample of participants investigated allowed us to reach a point at which no new categories emerged from transcript analysis. Thus, data saturation, defined as the point in data collection and analysis at which new information produces little or no change in the codebook [23], was achieved. 

Using patient-generated data via the interviews and verification of interpretation by a multidisciplinary group of researchers, we were able to assess the reliability of the data [30]. In short, as for credibility, confirmability and transferability, research methods were derived from previous comparable projects; familiarity with the culture and an adequate understanding of the participating groups of patients were developed before the first data were collected. Triangulation was used, with the final data analysis being confirmed by the multidisciplinary group of researchers (psychology, rheumatology, neurology, internal and pain medicine, physical therapy), so that findings emerged from a consensus between researchers. As pointed out by Barbour and Barbour [31], multidisciplinary research teams may raise challenges, but they allow data to be subjected to a range of ‘multidisciplinary gazes’ [32], drawing on a wider store of theoretical frameworks and insights, and “providing for a much more comprehensive and conceptually productive review than do traditional approaches based on triangulation with its restrictive focus on internal validation”. The key issue here was not the degree of concordance between researchers, but the insight provided by discussions for the refinement of coding frames [21]. Emergent findings were corroborated with existing theories and examined by comparison with previous research findings, to assess the degree to which they were consistent with the findings of previous studies. Finally, background data were provided, to ascertain the context of study and to allow comparisons to be made.

### Ethical considerations

All subjects were informed of the goals and design of the study and assured of confidentiality before formally agreeing to participate. Data were rendered anonymous to ensure confidentiality. The authors took care to conform to the ethical standards promoted in the Declaration of Helsinki. Institutional review board and French data protection agency approval (CCTIRS, CNIL) was obtained before subject enrollment. 

## Results

### Participants

Fourteen participants were included: 10 women and four men, aged 40 to 75 years. The sample included individuals with various professional qualifications. Seven respondents had diffuse OA, three had isolated knee OA, two had isolated hand OA, one had knee and hand OA, and 1 had isolated hip OA. Eight respondents were retired, one was on sick leave for disability, and five respondents were still actively working.

### Why it is so difficult for people suffering from OA to talk about pain and describe it?

The interview-reviewing process identified a major difficulty in getting arthritic people to describe their arthritis pains. Several reasons for this difficulty emerged from content analysis: the impossibility of comparing personal perceptions with a gold standard; the perception that nobody wants to hear about OA pains; the need to preserve one’s self and social image; the representation of OA; the notion of self-imposed stoicism; and the perception of OA as a complex, changing, illogical disease.

#### Inability to compare personal perceptions with a gold standard

This difficulty was related to the subjective experience of pain: “The difficulty in describing osteoarthritis pain is that I have never been ill. I’ve never felt pain, but I thought, I will try and work with what I know and that everybody has felt once in one’s life: toothache and labor and childbirth”. Therefore, when asked to describe OA pains, most respondents felt at a loss: how could they express, or describe, what they feel? 

It was even harder when the pain first began, with respondents tending to ignore OA pains, because they were not entirely sure that they existed at all. Indeed, these pains could be described as starting “low”, below the level of consciousness, so that when pain was not acute, but dull and constant, respondents generally reported mistaking the pain for a general state of “not being so well”: “It’s like a suspicion of pain more than a real pain, at the beginning. You don’t know that you are in pain, really. You just don’t stay there wondering: am I in pain or not? You just go ahead with your life, think of something else”. 

#### Nobody wants to hear about OA pains

This perception concerned doctors and nurses, according to the respondents: they either cannot or will not do anything about pain: “The physiotherapist said: that’s life!”. Often, uncaring audiences were both professional and friends and family. “My son doesn’t want to know if I’m not well. And of course he could do nothing about it. He says: “you will bury all of us! You are not really ill, you look fine!” What can I say?” And, when someone cared enough to notice, respondents would try to hide their pain, even deny it. Fear of giving pain to the ones they love and who care was then emphasized. 

The models the individual grew up with also contributed to this perception that nobody wants (or needs) to hear about OA pains: “Our grandparents had osteoarthritis. They never complained. I feel I owe it to them to be as brave and dignified as they were”.

Another added problem was that respondents sometimes talked about their pains but were not heard because nothing was visible on the X ray. The distrust displayed by healthcare professionals tended to make things worse. “Doctors say: ‘if you really had something, it would show on the X ray’. Nothing shows? Well, you’re all right then. You look crazy. People tell you it’s in your head, you’re a psychological case. So you’re in pain, and the way people look at you makes it worse!”

#### Necessity to preserve one’s self and social image

This necessity was expressed in various ways in the context of an illness seen as associated with age, loss of mobility, decaying autonomy and health. It stressed the manifold aspects of this dimension of self-image and included the preservation of social status, social life and relations with friends, as well as of self-image, and intimate image. As this patient indicated it: “I don’t talk about it to my wife because I always have the feeling that I exaggerate. I think I can be a bit of a hypochondriac, like most men, though I do think there really is something”. 

#### Representation of OA

Another reason for not talking about OA pains concerned the “natural” image of osteoarthritis. OA was felt to be a natural and inevitable phenomenon, related to aging, that neither patients nor doctors and science can fight, and not a matter of scientific research and progress. It nurtured a feeling of doom: “The fact that I came here and that we talk about it, I feel less isolated. I never talk about it to my colleagues, it’s an illness for the very old”. As such, it raised feelings of hopelessness, when faced to the first stigmata of age and the end of youthful health. “I work at an optician’s. Some women, who come for their first glasses around 40, they cry. Because they know they are getting… less young. I feel the same. I’m 40, I can’t run with my children, it’s over”. 

#### Self-imposed stoicism

Patients try to decrease the pain they feel by not thinking about it: this mechanism of dealing with pain was practiced by all respondents in this study. It was expressed in different ways: one was to insist on the importance of being optimistic: “I wonder sometimes if I exaggerate what I feel. One doesn’t live any more if you always think about your pain”; another way was to ignore pain, to decrease pain awareness so as to deal with the pain and go on with one’s life; and finally, in some instances, stoicism was induced by guilt, with overweight patients, for example, feeling “guilty” about their illness. They felt they “deserved” pain and should not complain about it, as this patient put it: “I also thought, this could be because I’m overweight, and the doctors insist on that, I’m 110 kilos, each of my knees manage 55. It’s a form of guilt that is imposed on us, of course it’s for our own good, we should lose weight, but vexation aside, it’s not that simple. I’m a good cook; I know how to cook light.” 

These examples show how self-imposed stoicism was part a show of inner strength and resilience, part autohypnosis. It may thus offer a double benefit: it improves self image, and effectively decreases the pain felt.

#### OA as a complex, changing, illogical disease

OA was described as not always feeling the same; its pains are different according to the moment of the day, the place where it hurts, or the weather. Variations were also described as unpredictable, with no clue as to their cause (a particular movement? the weather? an unknown physiological phenomenon within the body?). Moreover, older respondents emphasized that they had other diseases and therefore could not “really” know what was due to OA, and what was caused by another illness entirely.

In summary, respondents stressed the difficulties they experienced in talking about their pain, related to the absence of a clear concept to describe pain and of an accurate vocabulary: pain is not only a matter of intensity or duration. OA pains are numerous, and differ in respondents’ words in various respects, including painful sensations, type of occurrence, mode of initial impact, rhythm, and varied associated symptoms. It may also incapacitate the person totally or only partially and may be “noisy” or not. As this participant pointed out: “It is hard to describe, qualify my pain. There is this notion of orchestra: some bassoon, then along comes the clarinet”.

### Shades of pain

Despite this difficulty getting people suffering from OA to talk about pain and their feeling of a lack of accurate vocabulary to describe OA pains, content analysis made it possible to identify and list various characteristics of OA pains, some of which were common to most respondents (Table 2) and others that were less common (Table 3). OA pain characteristics could be grouped into seven dimensions: characteristics of pain sensation; pain variations according to the type of occurrence; triggering factors; physical activity; mood and representation of OA; OA-related symptoms; and general symptoms (Table 4).

**Table 2:** Core painful sensations expressed by the participants.

Name of pain	Main characteristic	Additional characteristics	Participants’ words
Background pain	Always there, located everywhere in the body	May be akin to long-lasting tiredness	*“It envelops your body, it’s like a cloud of pain, not strong but worrying, nagging, like a mosquito buzzing around”*
	Sort of oozing pain, with a dull pitch and a variable intensity	May start low and then become more present/ stronger, acute	
“Deep pain”	Inner location: close to or within the “bone”	Precise impact point hard to identify	*“Deep inside the body, not on the surface. It’s hard to know where it starts”*
	Dull to acute	Feelings of “alien things inside” = augmented anxiety	*“Like an alien moving in your body”; “a slow moving lava flow”*
“Stabbing pain”	Short, intense, brutally penetrating pain, with a slow, gradual withdrawal of pain	May happen at night. May stop as rapidly as it started. Tearing feeling / bodily integrity. threatened. Patient may stop moving/doing what he was doing with the “shock” of the pain. Sometimes cries, shouts. “Defective limb” feeling. Mostly in hands, knees.	*“Violent, occasional but regular. It doesn’t last long, only a few seconds. Deep. It will go away slowly, gradually”. “It can take up to one hour to ebb away”. “Pain stronger than anything else, 8/10”. “At the base of your thumb”. “In the knee when I turn my leg in a certain way. I’ve stopped going at the pictures because after 2 hours when you have to stand up, it’s awful”*
	May happen several times per day		
“Tearing pain”	Close to a stabbing pain but closer to the skin / with larger impact. Intense, acute pain; Lasts longer since it awakens the “background pain”	If very intense, feeling that “the heart will stop beating”. Anxiety provoked by intensity of pain. Seizes all of the body + awareness of patient. May happen at night	*“Like tearing something, you can’t trust your muscles anymore, as if I had bones only.” “I already stayed trapped by it 3 hours long in my bathtub because I couldn’t get up. Going on my knees is impossible to bear”. “ I already fainted from it”*
“Electrical shock”	Sudden, extremely acute, but less intense than the preceding pain.	No identifiable cause. Brief and goes away quickly, entirely. Global physical reaction to it. Some report a “vibrating” sensation. No regularity/pattern in occurrences.	*“Like touching a jellyfish”. “Intensity: 9/10, but doesn’t last”. “When it happens, I brutally stand up in the bus”; “you start up”. “Happens even if I’m not doing anything”. “Sometimes it happens 3 times in a short laps of time, sometimes nothing for some months”.*
	Less anxiety because no feeling of physically penetrating/tearing alien element.		
“Contact pain”	The merest touch awakens a fierce pain. Very common (most patients)	Very intense pain: “*unbearable*” that stops all movement (patient will drop what he/she was holding). Added tearing sensation.	*“You break a lot of plates that way”; “I will cry if I have to press a sponge”; “If I have to go on my knees - it’s as if I was kneeling on broken glass, it’s impossible, appalling. Las month I had to at my doctor’s, I shouted from the pain of it”. “This pain is 10/10”*
“Missing limb”	More discomfort + acute anxiety than real pain (not always a “pain”). Patient has the feeling his/her leg is suddenly missing or unable to carry him/her and is afraid of falling / really hurting himself/herself if on stairs, or outside, etc.	For some: violent pain (when caused by a movement?). Dull, pain when no particular movement involved. Deep pain. Feeling of unhinged limb. Arrested move. Loss of equilibrium.	*“Sometimes my leg will give way and I fall, so I have to be careful in the stairs”. “It’s abrupt, fast”. “It is a stress because you feel less assured. It’s a deep pain”. “You distrust you own body”. “Your knee feels like a sponge and the joint is not working anymore so you do not trust it and do not dare to put your weight on it”.*
“Cramp” (feels like) or wooden leg syndrome	(limb) Stiff under the touch + feeling of inner / outer tension.	A finger or a limb. Nearly crushing sensation (but not physical). Night or day; no identifiable cause. Dull pain; very large impact zone	*“As if the nerve was very tight and stretched to the maximum”. “A dull pain all along my leg, like a wooden leg”. “In the fingers, it is strange: they are stiff, with a contracted look”*
“Pulsating knee”	Dull pain – can be acute; burning “inside”, swelling around the knee	Warm to the touch. Pulsating pain + feeling prickling. Skin “too tight”, stiff. Red skin. Long: up to one day	*“I can feel my heart beating in my knee”. “Impossible to bend my knee. It’s twice as large as it was; warm to the touch, and painful. My skin is red too. Intense burning sensation, but it never lasts more than one day”*
“Brutal paralysis”	A “violent” pain, not lasting + limb/finger “blocked” + sensation of inner ravaging	May be noisy (cracking). Feeling of “inner ravaging”. Less frightening on knees. High level of anxiety when there is a feeling of ravaging/entropy inside.	*“Violent, but doesn’t last; fingers are contracted, you can’t put them down flat; it feels as if the cartilage is crumbling away, as if it were gravel inside”. “My husband hears it when it cracks, my grandchildren hear it when I am on the stairs”.*

**Table 3:** Less common pains.

“Crushing pain”	Physical sensation of “crushing” of a joint/limb	Mostly in hands, back. May come gradually or brutally. May be accompanied with fear for one’s body integrity. Deep pain	*“As if my hand was crushed, totally destroyed”. “A kind of liquefaction, as if your joint or cartilage was being liquefied” (one person only*)*; “Like a hammer blow”*
“Twisted fingers”	Acute pain in the fingers, with a feeling / physiological consequence of them being “twisted”	May affect the thumb only.	“Twisted fingers: no more rings for me”. “My thumb: I can’t open it anymore”
“Prickling”	Low intensity pain, more of an intense discomfort – a prickling sensation	Rhythmic pain (slow or fast). Close to numbness. Irregular, ‘illogical” occurrences, day or night. Larger impact zones	*“The 1001 stings”; “There is a rhythm to it, it can be fast or slow”. “A numbness, you don’t know what to do”; “like harassment”. “Comes back regularly, maybe because of a particular position?”*
“Burning pain”	Intense, acute pain akin to burning sensation	Rare (one occurrence). Deep pain (bone)	*“As if I were on the grill. Two grills inside, on both sides of my hips. That’s what I feel right now; a deep burning. The pain is deep inside; I don’t move much. If I turn round to watch you, my neck hurts; it doesn’t hurt if I do not move”*
Neck pain	Described as similar to migraine. Or: stabbing in the neck.	Location: back of the head/ shoulder paralysis; Associated headache; rhythmic pain. Or: stabbing pain in the neck + ravaging feeling	*“I stay in the dark, a small pillow under the neck. My head aches but at the back of my head”. “If I turn my head – frightening, as if there were gravel inside”*

**Table 4:** Seven dimensions identified through qualitative analysis.

**Dimension**	**Examples from the verbatim transcripts**
Pain sensation characteristics	I have burning pain. I have stabbing and crushing pain. Pain is like electric shock sensations
Pain variations	My pain increases at the end of the day. I have unpredictable peaks of pain
Pain-triggering factors	My pain is increased by wet and cold weather conditions
Pain and physical activity	My pain starts as activity starts
OA-related symptoms	My joint is stiff. My joint swells after exercise
Mood and image	My pain makes me feel isolated and old
General symptoms	My pain is exhausting me. I cannot sleep because of the pain

#### Characteristics of painful sensations

OA pains were often presented as very intense, with descriptions of violent, intense, brutal, acute, fierce pain. However, as noted above, OA pain is not only a matter of intensity and there are many different types of pain, described in the patients’ words, in terms of intensity, duration (from everlasting “background pain” to sharp, instantaneous “crises” brought on by a “bad movement”), or depth (muscle-deep vs. bone-deep). This sensation could develop into ‘abrupt paralysis’ combining pain intensity and anxiety related to the feeling of “being ravaged from the inside”. The importance of this perception of a physically penetrating/tearing element was further stressed by the presentation of ‘pain as an electrical shock’. Background pain was present in all patients, described as starting low and then becoming more present/stronger, acute. It was presented as a sort of oozing pain, with a dull pitch and a variable intensity. 

#### Pain variation according to the type of occurrence

“Starting pains” were described by all participants, with getting up (or standing up after a prolonged period in a sitting position) taking time because of pain. These pains were described as “mechanical”, a bit like trying to start up an old, rusty machine. Morning starting pains did not last long, but the sheer anxiety of it may make it feel longer: “When you unfold your knees, it’s 10 seconds of an awful pain. Then, it goes away. But you remember the pain”.

#### Pain triggering factors

OA pain was described as possibly related to external factors, including the weather in particular, with cold and wet weather conditions being the worst (hot and dry was a relief for the respondents), and bringing an extension of OA pains, both in intensity and in localization. The participants felt their whole body was “*seized*” by OA pain, and they described it as feeling like a “totally rusted” mechanical device (*“When it rains, it seizes up. I feel as if my whole body was jammed by osteoarthritis; it spreads. Like a robot with the whole body rusted”*). This last characteristic also added to the feeling that OA pains were ‘psychological’, i.e. whimsical, and therefore difficult to take seriously.

#### Impact of pain on physical activity

The first pain described by all participants was the background pain: the one you have to ignore to go on living, because it is always there. Patients tended to try and “tame” the pain by restricting their activities, their movements, or whatever they felt could “*awaken*” the pain and make it acute and bring it very much into the foreground.

#### Mood and representation of OA

Anxiety could be provoked by the intensity of pain (like in ‘tearing pain’), potentially leading to a feeling of threat against bodily integrity (as in ‘stabbing pain’). However, anxiety may also arise independently of pain intensity, as related to the feeling of “alien things inside” that ‘deep pain’ located close to or within the bone may create. Images of OA pain were frequently associated with older age and, in many cases, these pains were seen as the first sign of becoming old, and possibly isolated. 

### OA-related symptoms and local sensations

Various associated symptoms were described, such as a swollen knee, skin that was re, or warm to the touch, or too tight, and contracted limbs. A ‘missing limb’ sensation was often mentioned, presented as more of a physical discomfort than a pain, but causing anxiety nevertheless. 

#### General symptoms

OA pain may have repercussions for global behavior (some acute pains were described as possibly making you cry, shout, sweat or faint). As in most chronic pain, OA pain can be debilitating, exhausting and may cause sleep disorders. For some participants, background pain was akin to extreme, long-lasting tiredness. 

In summary, these seven dimensions highlight the major importance of pain sensations and descriptors. They highlight OA-related local symptoms, pain variations, triggers, physical activity and global physical repercussions of OA pain. They also emphasize the contribution of an emotional dimension. The “name of the pain” (Tables 2 and 3, 1st column), in the respondents’ own words, also reflected both the intensity of pain (with descriptors such as ‘electrical shock’ or ‘contact pain’) and its emotional dimension (e.g. ‘tearing pain’, or ‘alien inside’). This name of the pain, along with a description of pain characteristics highlighted the expression of both physical and emotional pains, as well as the extent of the various shades of pain.

## Discussion

### The need for a specific assessment of OA-related pain symptoms: pain is a complex sensation in OA, not adequately assessed by a single intensity rating

This qualitative study specifically aimed to qualify pain sensations and experience in people living with osteoarthritis pain. As shown in this study, it is difficult to get OA patients to relate their experience when asked to describe their pain. Several reasons for these difficulties have been identified, and the absence of a concept to describe OA pain and of an accurate vocabulary in terms of pain descriptors was highlighted. These findings support our hypothesis that patients find it difficult to express their complaints and that new tools are required to improve the qualification of pain sensations in OA. Despite the high frequency of OA, which is one of the most frequent painful conditions, very few studies have analyzed OA pains and the full range of sensations experienced by patients. By contrast to neuropathic pain, there are no specific questionnaires available for the analysis of sensory descriptions of OA pain and associated symptoms. Pain is a complex experience and a single-dimension pain rating is not suitable for individual assessment [33,34]. In neuropathic pain, several authors have developed specific questionnaires to improve the analysis of pain sensation, such as PainDetect [9], LANNS [8] and NPSI [7]. In osteoarthritis, some authors have tried to detect a neuropathic component through the use of Paindetect, and this has led to the development of a modified PainDetect questionnaire [35]. PainDetect has been studied in patients with OA, and this neuropathic component has been associated with signs of central sensitization [36]. This is consistent with our questionnaire, which includes several pain descriptors usually associated with specific features of neuropathic pain, and with certain studies demonstrating somatosensory abnormalities in knee OA, by quantitative sensory techniques [37]. 

Several composite questionnaires are used to analyze OA and its symptoms, including pain and associated disability. These questionnaires include, specifically, the Western Ontario and McMaster Universities Osteoarthritis Index [38], the Lequesne index [39] and the Arthritis Impact Measurement Scales [40].

Several questionnaires are suitable for more specific analyses of OA pain in populations of adult rheumatology patients: Pain ratings, McGill Pain short form, Short Form-36 Bodily Pain Scale and the recently developed arthritis-specific ICOAP questionnaire [41]. The ICOAP [12] is a multidimensional OA-specific tool developed jointly by OARSI and OMERACT for comprehensive evaluation of the experience of pain in people with hip or knee OA, including pain intensity, frequency, and impact on mood, sleep, and quality of life. Interestingly, qualitative analyses developed in the ICOAP study yielded results very similar to those obtained in our study, with nine categories of pain descriptors, and similar descriptors. However, the aims of this project were different, and the final ICOAP questionnaire does not provide pain descriptors, instead defining two types of pain (intermittent and background pain) and it aims to describe the most distressing pain features, in both hip and knees. It is thus more of a questionnaire for exploring the impact of knee and hip OA pain on quality of life than for specifically describing pain sensation in OA patients, with pain descriptors. 

### Barriers to talking about OA pain

The importance of helping patients and therapists to come up with ways of expressing (or understanding) the variety of OA pains was clear from the difficulty the OA patients had talking about their pain and describing it during the interviews. It is particularly important to provide initial encouragement to be candid and spontaneous in dealing with the subject at hand, and this encouragement should be reinforced and repeated, because respondents were initially reluctant to talk about “how painful exactly” their OA was. The respondents systematically required repeated assurances that such talk did indeed have a scientific aim and use, and that it would not be futile, or a “whining” about what they felt, but could actually help improve medical knowledge of OA. Trapped by stoicism, ideas about the nature of the disease (osteoarthritis comes with age, and nothing can be done about it), and the lack of pre-existing words or concepts that could be applied to it, respondents not only did not know how to describe their pain, but were also convinced that doctors were not interested and could do nothing about the pain in any case, as also reported by Jinks et al. [11] in a qualitative study on knee pain in older adults. 

The type of metaphor used provided further clues to the emotional/ psychological impact of pain. The respondents’ wording clearly stressed that pain was not limited to physical suffering, either in terms of its consequences or the actual experience of pain sensations, which also raised mental images and specific feelings [42]. Which is more intimately frightening: a pain like that when you inadvertently knock your knee or a “stabbing pain”, with the mental image of an alien intrusion within the body, or the feeling described as “being ravaged from the inside”, implying that some internal part of the body has broken down? In addition to the gradual scaling of physical pain (from light to intense), these descriptions of pain highlight the need to consider the emotional dimension, through the way this physical pain is read and decoded by patients [43].

### Better assessment of pain descriptors and associated symptoms in OA: treating OA-related pain according pain phenotype

The development of an instrument for improving the qualification of painful sensations in OA might help to improve the patient-tailored management of OA [44]. 

Pain treatments in OA are poorly effective, and outcome measures are based mostly on pain VAS or on questionnaires assessing pain purely in terms of its intensity, without taking into account its quality. In several pain conditions, different authors have developed questionnaires or more objective techniques, such as quantitative sensory testing (QST), for identifying predictors of treatment efficacy [45]. This may lead to the subgrouping patients according to their pain sensations and sensory disturbances, and the adaptation of treatment according to these characteristics, more accurately than could be achieved with a single pain intensity measurement. We believe that a questionnaire on pain quality, with pain descriptors, is an essential complement to the ICOAP questionnaire developed by OARSI/OMERACT. ICOAP is a questionnaire focusing on the patient’s priorities, defining constant or intermittent pain conditions, whereas our qualitative questionnaire, in which most pain descriptors are grouped into dimensions, may help to define OA pain phenotypes. This type of analysis, with the definition of descriptors, has been reported for neuropathic pain conditions with the NPSI [7], and should be developed in OA pain. 

### Development of better communication, to improve compliance with treatment

Identifying patients’ representations and taking them into account is also a key issue in the patient-centered management of OA, which aims to improve patients’ knowledge of the disease and their compliance with treatment [46,47]. In osteoarthritis, a lack of agreement between clinician and patient priorities can affect the clinician-patient relationship, compliance with treatment and potential health outcomes [48]. In this context, identifying a vocabulary that considers the patients’ description and is accurate enough to allow congruent communication between health professionals and patients should make this information more effective [49]. For example, the identification of patient-reported clinically important outcomes may improve post-surgery follow-up [50].

### Study limitations and strengths

This study provides insights into the types of pain experienced by people with osteoarthritis, but the limitations of its design and outcomes should be recognized. In particular, as in many studies, the transferability of the findings is limited. As in all qualitative studies, our study sample was small and transferability may thus be limited to people and settings with characteristics similar to those investigated in this study. Moreover, exploring the experiences of individuals with OA pain and the meanings they attribute to these pains requires recall, and the participants’ responses may thus be limited by recall bias. 

This study was based on interviews with a diverse group of respondents on OA pain experience, an apparently common topic that is, however, rarely investigated from the patients’ point of view, i.e. using the respondents’ own description of how they perceive and describe pain and the related changes. The focus groups were conducted by a psychologist who was not a healthcare provider and had no prior relationship either with the respondents or with the multidisciplinary team before data collection, to try to minimize the risk of overlooking responses too close to or far from the expectations of healthcare providers. We convened the multidisciplinary group of researchers (psychology, rheumatology, neurology, internal and pain medicine, physical therapy) to review the findings, to minimize the risk of missing important contextual information in the focus groups and to contribute to the understanding of clinical situations [51].

## Conclusions and Implications for Future Research and Clinical Practice

It is common for people to normalize joint pain and not to consult a doctor, and our patients’ interviews help to understand why people do not speak about their OA pain. Even if patients with OA pain do not express their pain spontaneously, this study demonstrates that pain should not be analyzed solely in terms of its intensity, because many painful sensations and associated symptoms were reported by patients with osteoarthritis. 

Our qualitative research led to the definition of seven dimensions in OA pain. Based on these dimensions, we aim to develop a specific questionnaire on OA pain quality, for OA pain phenotyping: the OsteoArthritis Symptom Inventory Scale (OASIS). This new questionnaire, based on patient-reported experiences of pain, will be in line with IMMPACT recommendations, which promote the use of patient-reported outcome measures in the assessment of pain and pain management [52]. Subgrouping patients with OA pain on the basis of pain quality, as assessed by patient-centered questionnaires, may facilitate the development of more accurate and effective pain management. We will also investigate whether any particular dimension is associated with a different pathophysiological mechanism, such as nociceptive, neuropathic and central sensitization. 
